# Dentoskeletal Stability in Conventional Orthognathic Surgery, Presurgical Orthodontic Treatment and Surgery-First Approach in Class-III Patients

**DOI:** 10.29252/wjps.7.3.283.

**Published:** 2018-09

**Authors:** Massoud Seifi, Negin-Sadat Matini, Amir-Reza Motabar, Mahtab Motabar

**Affiliations:** 1 Department of Orthodontics, Dental School, Shahid Beheshti University of Medical Sciences, Tehran, Iran;; 2 Shahid Beheshti University of Medical Sciences, Tehran, Iran;; 3 Department of Plastic Surgery, Bou-Ali Hospital, Azad Medical University, Tehran, Iran;; 4 Department of Periodontology, Dental School, Tehran University of Medical Sciences, Tehran, Iran

**Keywords:** Surgery-first, Orthodontics, Orthognathic surgery, Conventional approach

## Abstract

For many years, the conventional approach to orthognathic surgery which was orthodontic treatment prior to orthognathic surgery has been the accepted method of treatment for skeletal class III malocclusion patients. This review compared the dentoskeletal stability of treatment results between conventional orthognathic surgery methods with presurgical orthodontic treatment and surgery-first approach in skeletal class III patients. The study protocol was based on Preferred Reporting Items for Systematic reviews and Meta-Analyses (PRISMA) statement for systematic review and meta-analysis. Electronic and manual searches for literature since 2011 were conducted. PubMed and Medline databases were accessed. Data extraction and analysis were performed by two independent individuals. Seven studies out of hundred-fourteen articles met the inclusion criteria and were selected for qualitative analysis. The included studies were 494 patients with skeletal class-III malocclusion. Stability of treatment was compared between surgery-first approach and conventionally treated patients. The statistical analysis confirmed that surgery-first approach did not show more stability compared with presurgical orthodontics. The surgery-first approach shortened the overall treatment duration. However, more skeletal stability in conventional treatment was assessed. Both surgery-first approach and conventional treatment with presurgical orthodontics resulted in favorable skeletal changes in class-III malocclusion patients. Moreover, these findings should be discussed further due to the variety of study designs, outcomes and biases. Current evidence in this field still needs to be expanded. The authors wish to see more well-designed randomized controlled trials with long-term follow ups to confirm the results.

## INTRODUCTION

For many years, the conventional approach to orthognathic surgery which include orthodontic treatment prior to orthognathic surgery has been the accepted method of treatment for skeletal class III malocclusion patients.^1^ However, there are some limitations such as long duration of the treatment and worsening of patients profile during pre-surgical orthodontics which decrease patient’s compliance.^2^ The surgery-first approach have also been introduced as an alternative for the mentioned treatment plan. By the surgery-first approach, the need for any major orthodontic treatment preoperatively is diminished.^3^^,^^4^

In the conventional approach, dental decompensation is the result of presurgical orthodontics. In Class III malocclusion, the dental compensation usually evolves buccolabial flaring of the maxillary dentition and lingual tilt of mandibular dentition that will develop less class III appearance for patients and longer treatment time (for dental decompensation) with skeletal class III and mandibular prognathism.^5^ This probably is a disadvantage of the conventional approach due to less satisfaction of the patient.^6^

Through recent years, the surgery-first approach has become a popular treatment plan for patients regarding the factor that it may decrease the treatment duration by the reduction of preoperative orthodontics. This approach may lead to improved cooperation and has shown patients satisfaction with regards to immediate improvement of the facial profile or upper airway constriction.^7^^-^^9^ Also, increased tooth movement has been shown due to regional acceleratory phenomenon.^10^ It is reported that surgery-first approach can be performed in cases with mild crowding and proclined/retroclined anterior teeth, mild to moderate curve of Spee, mild vertical problem, and little or no transverse discrepancy.^11^

Although these advantages of surgery-first approach have led to acceptance of this treatment plan toward patients, it has not been fully investigated that this approach is more stable in post-operative occlusion compared with conventional approach.^12^ Proper dentoskeletal stability maintain more permanent results of the treatment. So it must be considered before a surgical method in order to ensure less relapse.^13^ Moreover, there is still no clear evidence regarding the stability of the results between surgery first and conventional approach under unstable post-operative dental and skeletal position.^14^ This study compared dentoskeletal stability between conventional orthognathic surgery, presurgical orthodontic treatment and surgery-first approach in skeletal class III patients.

## MATERIALS AND METHODS

*Search Strategy*

An Electronic search of PubMed was performed from 2011 to 2016. Based on the PICOS (participant, intervention, comparisons, outcome, and study designs), the inclusion criteria were non-syndromic adult patients with skeletal class III malocclusion who were treated with surgery-first approach or conventional approach. The outcome was to assess the post-operative stability between both approaches. Studies which had the least level of evidence, i.e. case reports were not included in the criteria. The search strategy was defined with the sequence as the following keywords: (“surgery first”) AND (“orthodontics”) OR (“orthognathic surgery”) AND (“orthodontics”) AND (“stability”). All publications were restricted for language and only English literatures were included. 

**Fig. 1:**
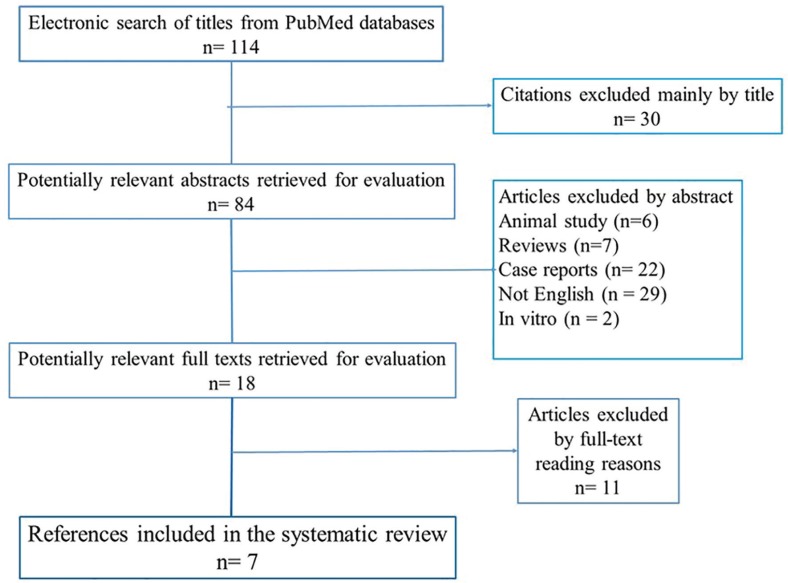
PRISMA diagram for search strategy in systematic review

The exclusion criteria contained articles done on animals and in-vitro designs, case reports and reviews, studies referring to orthognathic surgery and orthodontics without determination of post-operative stability. The systematic review was also based on Preferred Reporting Items for systematic reviews and meta-analyses (PRISMA) diagram guidelines. A description of electronic search is provided on Figure 1. In details. Two independent authors performed eligibility assessment and the screening of title and abstracts. Any discussion regarding full-text selection was discussed with a third reviewer. Another different author checked the random selection of filtered articles. After the acceptance of each abstract articles the authors read the full-text and included each full-text according to inclusion and exclusion criteria.

*Data Analysis*

Before carrying out the analysis and pooling the results, the heterogeneity hypothesis was tested using Chi-Square test. Then estimated effect size, the mean difference of change after intervention between two treatments were pooled with adjustment for heterogeneity by using random effect model meta-analysis. The graphical display of these estimated results are shown in forest plot.

## RESULTS

A total of 114 abstract were identified from the databases. However, only 7 articles matched the criteria. The PRISMA diagram gave a selection review of the search sequence. Table 1 summarized all included articles in the systematic review. Methodology quality could not be assessed with Cochrane Collaboration as none of the studies were clinical trials. The contents of the selected full-texts determined post-operative stability of the treatment by the comparison between lateral cephalometry analysis prior and after procedures. The excluded articles did not mention stability results of the treatment. 

**Table 1:** Summary of included articles in systematic review

**Authors and year of publication**	**Origin**	**Study type**	**Sample size and distribution**		**Mean age at the time of surgery**	**Aim of study**	**Type of intervention**	**Total treatment time**	**Stability**
Ko et al., 2013	Taiwan	Retrospective, cohort		N=45 (19 male, 26 female)	23.2	Identifying the parameters related to skeletal stability after orthognathic surgery in skeletal class III malocclusion using SFA and to analyze the factors correlated with relapse	Bimaxillary surgery; lefort I osteotomy, BSSO, and genioplasty (the latter only in 22 subjects)	13.9	At debonding, 12.46% relapse. Mean B-point relapse, 1.44
Choi et al., 2015	South Korea	Prospective, case control	N=56 (16 male, 40 female) 24 CA, 32 SFA		22.4	Clarifying that OGS without presurgical orthodontics may be effective as the Conventional approach(CA) in correcting dentofacial deformities	Bimaxillary surgery: Lefort I osteotomy with posterior impaction of the maxilla and BSSO or mandibular setback	SFA 19.4 CA 22.3	At 12 to 36 months follow-up relapse rate was not statistically significant between groups except for the lower anterior facial height ratio
Kim et al., 2014	South Korea	Retrospective, cohort	N=61 (28 male, 33 female) 38 CA, 23 SFA		CA 21.6±3.5 SFA 23±6.3	to compare the surgery first approach (SFA) with Conventional approach in terms of stability after mandibular setback in skeletal class III subjects	Mandibular surgery: BSSO	SFA 15.4 CA 22.5	At debonding, mandible moved anterioinferiorly. Average amounts of anterior relapse, 1.6 mm in the CA group and 2.4 mm in SFA group. Vertical relapse pattern was similar, Relapse <1.5 mm more dominant in CA. significant association between degree of relapse and group difference.
Y k Kim et al., 2014	South Korea	Retrospective, cohort	N=12 (5 male, 7 female)		19.83±2.37	Evaluate the association between the transverse changes of arch dimension and postsurgical relapse of the mandible after mandibular setback with minimal orthodontic preparation without extraction	SSRO	19.43±2.37	The changes in arch width had no association with horizontal and vertical relapses of the mandible.
Park et al., 2014	South Korea	Retrospective, case control	N=60 (24 male, 36 female) 36 CA, 24 SFA		CA 22.4±4.4 SFA 22.4±4.6	Comparison of SFA with CA in the amount and pattern of maxillary incisor inclination change in skeletal class III treated with extraction of the maxillary 1^st^ pm and bimaxillary surgery	Bimaxillary surgery: Lefort 1 osteotomy + BSSO. Maxillary 1pms were extracted during surgery in the SFA group	NR	NR
JY Kim et al., 2014	South Korea	Retrospective, case series	N=37 (10 male, 17 female)		23±4	Evaluation of postoperative stability of the treatment of mandibular prognathism treated with the SFA (IVRO)	Bimaxillary surgery: Lefort I osteotomy+ IVRO	14±6	No significant changes were observed in maxillary position after 1 year. The mandible had no significant relapse horizontally, but vertical relapse was significant at all time intervals, especially during the first 6 months postoperatively. Both anterior and posterior facial heights were decreased 1 year, and most changes occurred during the first 6 months postoperatively.
Park et al., 2016	South Korea	Retrospective, case control	N=40 (25 male, 15 female) 20 CA, 20 SFA		CA 25.25±3.77 SFA 22.60±5.39	Comparison of postoperative stability following bimaxillary surgery performed either with or without preoperative orthodontic treatment	Bimaxillary surgery: Lefort 1 osteotomy + BSSO	14.7	No statistical differences were observed in the relapse rate between the two groups.

A total of 311 patients with the mean age of 22.5 with the range of 18 to 37 were included. The sample size were not less than 37 and the maximum sample size was 61. Four studies reported Lefort I osteotomy with the addition of Bilateral Sagittal Split Osteotomy (BSSO), while 1 study only mentioned BSSO as surgical intervention. Occlusal stability was assessed by cephalometric analysis. Serial lateral cephalometric radiographs were obtained pre and post operatively. In order to compare dentoskeletal stability changes between selected articles, similar cephalometric parameters were identified. These 6 measurements included Sella-Nasion to A Point angle (SNA) which indicates the relative anterior-posterior position of the maxilla in relation to cranial base, Sella-Nasion to B Point angle (SNB) indicates the relative anterior-posterior position of the mandible in relation to cranial base, (B-point) the innermost point on the contour of the mandible between the incisor tooth and the bony chin, Overbite, Overjet and Angle between long axis of lower incisor and mandibular plane angle (IMPA). 

**Fig. 2:**
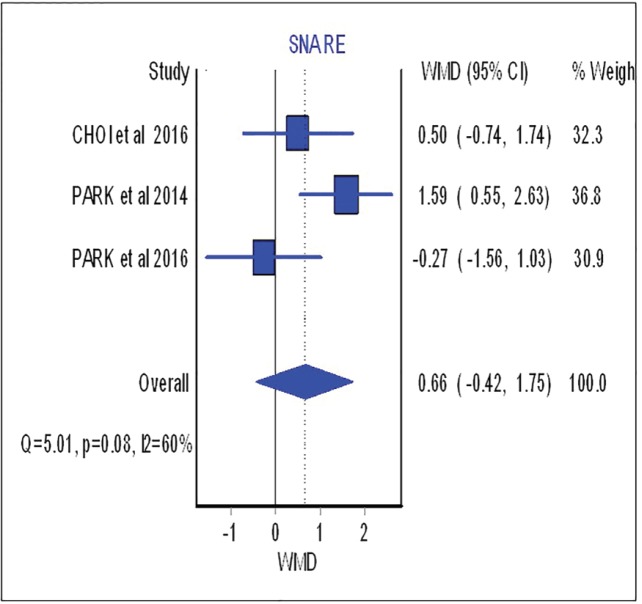
Forest plot analysis for SNA

Overbite and overjet were commonly introduced as parameters for measuring occlusal stability. Hence, SNA, SNB, B-point and IMPA were included to demonstrate skeletal stability before and after the surgical procedure. Based on meta-analysis, there was no significant difference between occlusal stability of surgery-first approach compared with conventional treatment. These analyses were able to determine post-operative stability in both horizontal and vertical planes. The forest plot with mean difference of change after treatment and 95% CIs and the pooled estimates for mean difference of change of SNA were illustrated in Figure 2. 

**Fig. 3:**
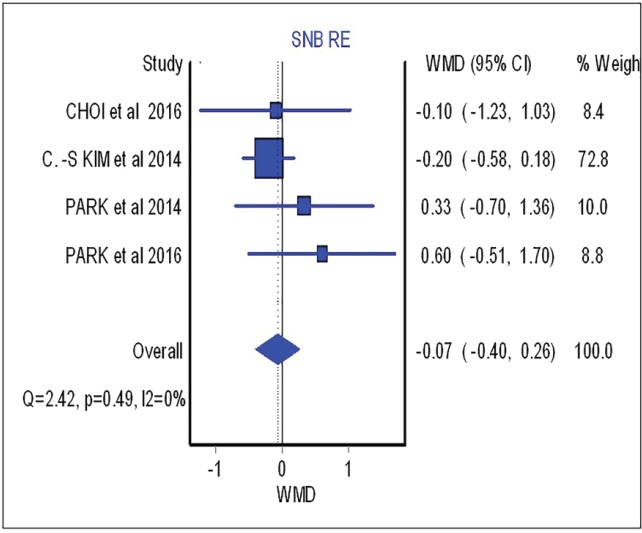
Forest plot analysis for SNB

**Fig. 4:**
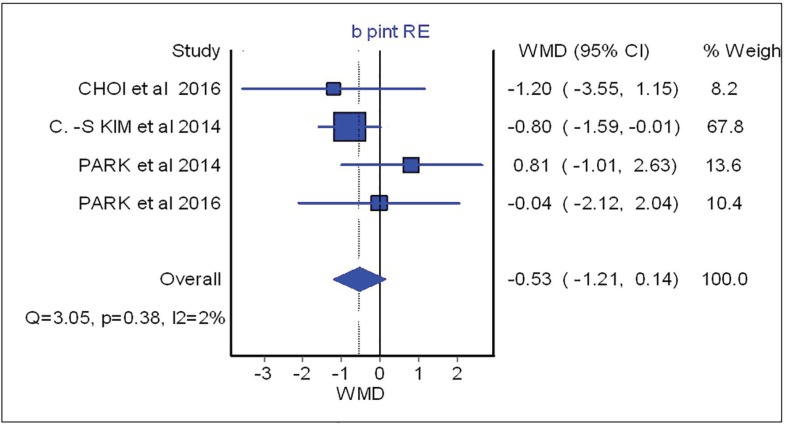
Forest plot analysis for B-point

**Fig. 5:**
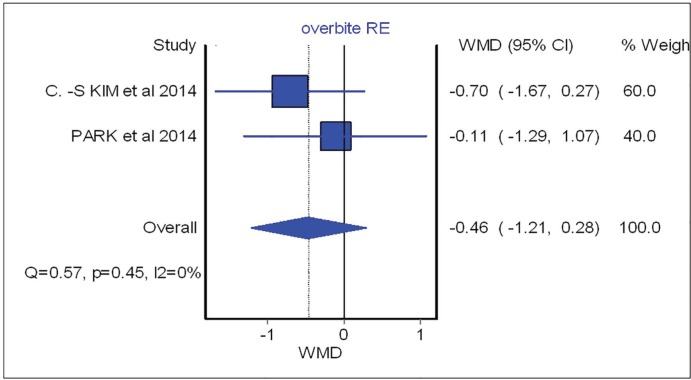
Forest plot analysis for overbite

**Fig. 6:**
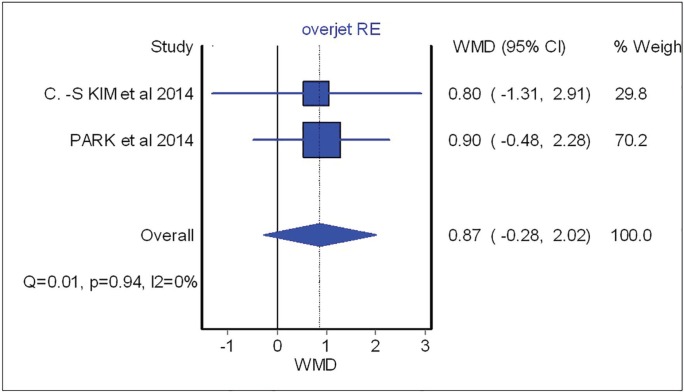
Forest plot analysis for overjet

**Fig. 7:**
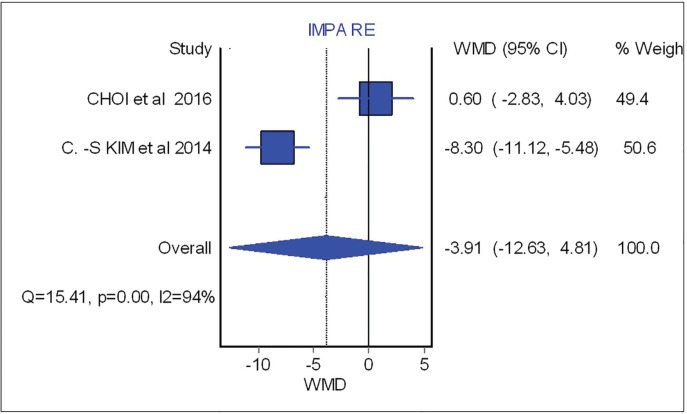
Forest plot analysis for IMPA

As shown in this figure three studies assessed the SNA changes before and after the surgical procedure. However, neither showed significant difference between surgical-first and conventional approach with regards to SNA changes (overall: weighted mean difference (WMD)=0.66; 95%, CI=-0.42, 1.75). Figure 3 (overall: WMD=-0.07; 95%, CI=-0.40, 0.26) demonstrated four studies comparing SNB changes between two different treatment approaches, SNB had no significant difference in both mentioned treatments as the same result was shown for other parameters such as B point (overall: WMD=-0.53; 95%, CI=-1.21, 0.14), overbite (overall: WMD=-0.46; 95%, CI=-1.21, 0.28), overjet (overall: WMD=0.87; 95%, CI=-0.28, 2.02), IMPA (overall: WMD=-3.91; 95%, CI=-12.63, 4.81) as are shown in Figure 4, 5, 6 and 7, respectively. Based on the meta-analysis, there was no significant difference in occlusal stability between surgery-first approach and the conventional approach based on the measured parameters.

## DISCUSSION

This study included 3 retrospective cohort,^12^^,^^15^ 1 prospective case control,^16^ 2 retrospective case control,^17^^,^^18^ and 1 retrospective case series.^19^ Four studies reported Lefort I osteotomy with the addition of BSSO,^12^^,^^16^^-^^18^ while 1 study only mentioned BSSO as surgical intervention.^15^ In another study, patients underwent a Sagittal Split Ramus Osteotomy (SSRO)^20^ and the other study was a combination of Lefort 1 osteotomy and Intra Oral Vertical Ramus Osteotomy (IVRO).^19^ Ko *et al.* included genioplasty among other interventions.^12^ Ko *et al.* used monocortical plates and screws in each side of mandible and miniplates in each side of maxilla as post-surgical fixation.^12^

In Kim *et al.*’s study, mandible was fixed with 4 miniplates in a monocortical fashion and intermaxillary elastics and also interocclusal splint were given to patients 4 to 6 weeks after the procedure.^15^ Two weeks of archwire and 8 mini implant were used as an intermaxillary fixation*.*^20^ Choi *et al.* also used mini plate as internal rigid fixation.^16^ However, in another study arch wires were used for 2-3 weeks as surgical fixation.^19^ Park *et al.* used metal plates and screws for fixation of mandible and a maxillomandibular fixation with surgical stent including ball clasps that were applied for 5 days in order to achieve retention.^18^

In the study by Ko *et al.*, no presurgical orthodontics was performed in any patients. Kim *et al.* positioned inactive brackets for surgery-first group and had a presurgical orthodontic treatments included leveling, alignment and elimination of crowding for conventional approach group.^15^ In study by park *et al.*, all patients in both surgery-first and conventional group received non-extraction orthodontic treatment for mandible and the maxillary premolar was extracted during the surgery for surgery-first group patients.^17^ Choi *et al.* positioned inactive braces 1 month prior to procedure due to intermaxillary fixation in conventional approach, also the presurgical orthodontics provided decompensation of teeth axes, and coordinating upper and lower arches.^16^

Two studies have assessed the stability after 6 months of follow up,^18^^,^^20^ however three studies have indicated a 12 months of post-operative examination^12^^,^^16^^,^^19^ and one study analyzed the patients after 22 months of follow up.^15^ Recently, surgery-first approach has been introduced in various surgical and also orthodontics publications. However, few studies have compared this approach with the conventional orthodontic-first approach in treatment of patients with skeletal class III malocclusion. Numbers of studies with clinical trial design for the mentioned subject is still rare.^21^ Despite the lack of relevant publication, surgery-first seems to have a shorter treatment time.^7^^,^^19^^,^^22^ The efficient orthodontic procedure in surgery-first approach is reported to be in relation with regional acceleratory phenomenon (RAP) which is the result of transient demineralization of the bones caused after surgical procedure.^23^^-^^25^

Our aim in this systematic review was to compare the difference between surgery-first approach and the conventional approach in details. Dentoskeletal relapse is reported to have association with factors such as fixation method of bone plates, muscle contraction, maxillary constriction, curve of Spee, greater overbite and overjet. Better stability was exhibited in patients with flat curve of Spee and smaller overbite.^12^ Hence, some authors reported that its occurrence may be related to the surgical approach and the sequence of orthodontic treatment with surgery. Based on dentoskeletal stability, in surgery-first approach, better arch coordination can be resulted after the elimination of functional muscular forces in disproportional skeletal situation.^26^

After surgery, muscular loads will equilibrate and establish in proper position of teeth relative to apical base of the jaws and the jaws position will be more favored with less treatment duration. For instance, in class III mandibular prognathism, lower lip and mentalis muscle pressure against lower anterior teeth is a resisting functional load versus aligning orthodontic force. In disproportion skeletal situation, these contestant strains produce deleterious effects such as bone resorption, bone dehiscence, root resorption, and increased inflammation due to prolonged treatment time.^26^ When bringing jaws into appropriate position, a more balanced muscular force will be established. In the latter situation, orthodontic forces for alignment and leveling of teeth work optimal which means back-and-forth and jiggling movements will be avoided. In transverse dimension, force of buccinators muscles, or so called the buccinator mechanism [upper and lower buccinator fibers, superior constrictor muscles (attached to pharyngeal tubercle), upper and lower orbicularis oris muscle fibers] is exerted to the maxillary and mandibular posterior teeth resulting linguoversion of them.^26^

This condition happens when perioral muscular force conquers the muscular force of the tongue. In rare cases, tongue force overcome the perioral muscular strain resulting outward positioning of the posterior teeth. These facio-lingual positions that are described as “torque” of teeth should be corrected in order to produce ideal interdigitation of dentition which is perquisite for occlusal stability. A moderate level of faciolingual discrepancies is consistent with Surgery-First Approach (SFA)s. In Conventional Approach (CA), the mentioned discrepancies are never the less corrected by less limitations regarding complex skeletal problems. Again, in transverse dimension; contestant forces act as vying loads in contrast original situation versus corrected torque and adjusted posterior teeth relative to apical base.^17^^,^^20^

As in vertical dimension, surgery-first is able to position the tongue in a more proper relation with occlusal level, so less muscular resistance will be resulted for the later orthodontic treatment. According to the available data and authors’ studies, majority of skeletal and dental relapse takes place within short period after releasing inter maxillary fixation (IMF) postoperatively and initiation of the jaw function i.e. two to six months. The minimum follow-up period was 6 months in only two studies among all included studies in the present review. Based on the instability studies, it seems that dental and skeletal relapse are inevitable phenomena. Biologic boundaries are inherent limitation to the orthodontic tooth movement and skeletal repositioning.^27^

Apart from the mentioned general reason, surgery-first approach is a technique sensitive procedure. Even a highly experienced orthodontist/surgeon cannot clearly predict of the ideal post-treatment occlusal relationship. Improper fixation of the jaw pieces, malunion of healing osteotomized bone segments, loose IMFs (immediate or delayed loosening of the screws), incorrect position of the condyles relative to cranial base i.e. glenoid fossa, improper placement of the proximal/distal segments which leads to torqueing of the proximal parts, and inappropriate splint are among the factors that are noticeable in a technique sensitive orthognathic surgery.^15^

 In addition to the consideration of the mentioned factors in skeletal stability, centric occlusion–centric relation (CO-CR) discrepancies that are related to stability and function have a pivotal role in a sustained equilibrium of the functional matrix. Dental etiologic factors as a basis for relapse such as unstable position of the teeth regarding the biologic boundaries i.e. placement of the teeth outside of the jaw base functional matrix should not be taken for granted. Post-operative stability has not been fully investigated yet between these two approaches. Also, previous studies could not define precisely between different stabilities terminology. Authors of this review note that every skeletal instability leads to occlusal instability however, every occlusal instability is not equal to skeletal instability necessarily. Future caution must be taken to not confuse skeletal stability with occlusal stability and not to assess them with one another in upcoming clinical trials and case studies.^15^ Prior to the current study, it was unclear that which approach has gained more stability after the procedure. 

However, lack of patients and highly-qualified study designs are the limits in this systematic review. Another limit is related to population which all included studies were assessed. All included researches were applied in Asian population and were not performed on other populations such as Caucasians or Africans. So authors suggested alike studies on a diversion of population in order to investigate the stability outcomes of both approaches in patients. As it is explained in previous articles, the most definitive advantage of surgery-first approach is the shortening of treatment duration. However, by eliminating presurgical orthodontics, the surgeon may need to apply more invasive procedures. On the other hand, surgery-first approach may not be performed on patients with complex orthodontic deformities. Some articles reported the reliability of wearing splint in achieving a stable dentofacial position after surgery-first approach.^15^

Kim *et al.* reported the positioning of the maxilla as an important risk factor for mandibular relapse however the results showed same relapse in both explained surgical methods.^15^ Accelerating tooth movement is another aspect which may result rotational relapse.^11^ In addition to previous studies and the present systematic review, it was shown that long-term follow ups and a large homogenous samples are crucial in order to evaluate more exact arch dimensional changes. As the matter of dentoskeletal stability, no significant difference was shown between surgery-first approach and the conventional orthodontic-first approach for class III malocclusion patients. Practitioners are able to suggest both treatment plans as an appropriate method based on other occlusal and skeletal parameters.
